# Determinant of emergency contraceptive practice among female university students in Ethiopia: systematic review and meta-analysis

**DOI:** 10.1186/s40834-020-00123-8

**Published:** 2020-10-05

**Authors:** Rekiku Fikre, Belay Amare, Alemu Tamiso, Akalewold Alemayehu

**Affiliations:** 1 Department of Midwifery, Hawassa University, College of Medicine and Health Sciences, P.O. Box 1560, Hawassa, Ethiopia; 2 Hawassa University, College of Medicine and Health Sciences, School of public health, P.O. Box 1560, Hawassa, Ethiopia

**Keywords:** Emergency contraceptive, Ethiopia, Female university students, Systematic review, Meta-analysis

## Abstract

**Introduction:**

Despite Ethiopia’s government’s commitment to alleviating unwanted pregnancy and unsafe abortion by increasing holistic reproductive health service accessibility, the rate of unwanted pregnancy among female students in the universities is distressing and becoming a multisectoral concern. Therefore, this systematic review aimed to assess the prevalence and determinant of emergency contraceptive practice among female university students in Ethiopia.

**Result:**

The overall pooled prevalence of emergency contraceptive practice among female university students in Ethiopia was 34.5% [95% CI [20.8, 48.2%]. The pooled odds ratio showed that positive association between practice of emergency contraceptives with age of the students [OR, 0.19; 95% CI: 0.04, 0.98, *P* = 0.05] Previous contraceptive methods use [OR, 0.22; 95% CI: 0.12, 0.40, *P* = 0.0001], Marital status [OR, 0.09; 95% CI: 0.02, 0.40, *P* < 0.002] and knowledge [OR, 0.12; 95% CI: 0.04, 0.37, *P* < 0.0003].

**Conclusion:**

The practice of emergency contraceptives among university female students was 34.5% and explained by knowledge, age, previous use of contraceptive methods and marital status.

## Background

Worldwide, 250 million pregnancies are occurred annually, and 11% of pregnancy are accounted by adolcent then, one third of them are untended and 20 % of the pregnancy ended up with induced abortion [1, 2].

The Young generation was facing multiple reproductive health problems and among them, unintended pregnancy poses a major contest in developing countries. Due to economic dependability and lack of friendly approach in the facility, young women prone to end unwanted pregnancy through unsafe conditions which take the highest share for morbidity and mortality compared with adult women [3].

Around 80 million unintended pregnancies occurred in the developing world in 2012, resulting in 40 million abortions and 10 million miscarriages [4]. According to the World Health Organization report every year, nearly 5.5 million African women have unsafe abortions. Moreover, 59% of all unsafe abortions in Africa are among young women aged 15–24 years [5].

In the Ethiopian context, Emergency contraceptives are not part of family planning methods but used as an emergency contraceptive by women when they encountered different situations that predispose them for unwanted pregnancy [6]. Even though the practice of emergency contraceptives was low in Ethiopia, Emergency contraceptives can reduce the risk of unintended pregnancy by 75 to 99% if it is taken within three days of sexual intercourse [7, 8]. The impact of emergency contraceptives on the prevention of unplanned pregnancy and to avoid unsafe abortion which is a treat for young women were deceived [9].

Several studies revealed that the practice of emergency contraceptive is different from one country to another. The practice of emergency contraceptives was (28%) among South African university students [10], (7.4%) in Cameroon [11] and (5.4%) in Nigeria [12].

The Ethiopian demographic health survey 2016 (EDHS 2016) report showed that contraceptive prevalence rate among Ethiopian women aged 15–49 is 36%, however, the practice of emergency contraceptives among sexually active unmarried women is low 4% [13].

Planned Pregnancy is a period of transition from childhood to an adult but if it was unplanned, the life of young women could have changed in many ways making them vulnerable to poverty and exclusion, and their health often suffers [14].

A study showed that the practice of emergency contraceptives in Ethiopia is below 10% [15, 16]. The magnitude of emergency contraceptive utilization practice among female University students in Ethiopia ranges from lowest 4.9% to the highest 78% [17, 18].

Therefore, this study aims to summarize evidence of emergency contraceptive practice among female university students in Ethiopia.

## Methods

### Search strategies and quality appraisal

The protocol for this systematic review and meta-analysis has been enrolled in the International Prospective Register of systematic reviews (PROSPERO). The methodology of this systematic review and meta-analysis was developed by following the Preferred Reporting Items for Systematic Reviews and Meta-Analyses (PRISMA) Additional file one.

The authors conducted systematic literature searches from the authentic major electronic databases such as MEDLINE, PubMed, EMBASE, Emcare, CINAHL (EBSCOhost), Web of Science, Scopus, Poplin, and Google Scholar. Also, the hand (manual) accomplished to retrieve unpublished studies and gray literature. We used MeSH terms, key terms, and search engines by extracting from the review questions for all the searches. The search strategy included “Predictors” OR “Determinants” OR “Related factors” OR “Factors” AND “Emergency contraceptive practice” OR “Emergency contraceptive utilization” OR “Emergency” AND “Practice” AND “Ethiopia”. Both authors constructed the search strings (RF and AA). The overall search result was compiled using EndNote X9 citation manager software Additional file two.

Later, articles were screened through a careful reading of the title and abstract. The two authors screened and evaluated the studies independently. The titles and abstracts of studies that mentioned the outcomes of the review were considered for further evaluation to be included in the systematic review and meta-analysis. Then the full-texts of the retained studies were further evaluated based on the quality of their objective, methods, participants/population, and key findings. The authors (RF, AT, and BA) independently evaluated the quality of the studies included against the Joanna Briggs Institute (JBI) critical appraisal tool checklists.

**Fig. 1:**
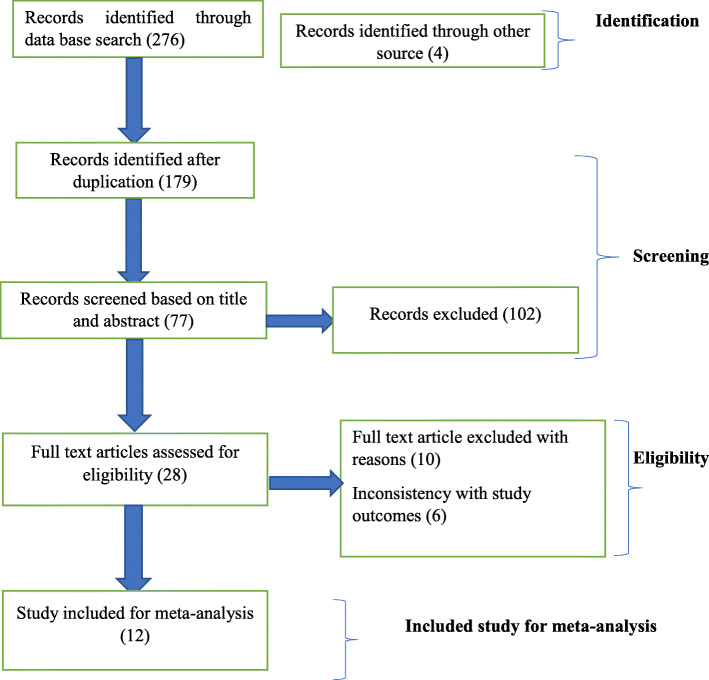
Description of a schematic presentation of the PRISMA flow diagram to select and include studies, 2020

In case of disagreement between the quality assessment results of the two authors, the differences were resolved by consensus for inclusion. The overall study selection process is presented using the PRISMA statement flow diagram (Fig. 1).

### Data extraction and analysis

Findings from the selected studies were extracted and stored using data extraction template prepared on Microsoft Word and then to Excel (2016), followed by extraction of important data based on study characteristics (first author, year of publication, study design, and outcome of interest) by the two authors independently. Meta-analysis was conducted using OpenMeta and CMA version 2 software to compute the pooled prevalence and factors associated with the emergency contraceptive practice.

### Heterogeneity and publication bias

Heterogeneity between the included studies was examined using the *I*^*2*^ statistic. A meta-analysis of observational studies was conducted, based on recommendations made by Higgins et al. (An I^2^ of 75/100%, suggesting considerable heterogeneity).

## Result

### Review studies

A total of 276 articles were identified through the major electronic databases and other relevant sources search from January 1/2020 to Febraruary1/2/2020. From all identified studies, 179 articles were removed due to duplication while 77 studies were reserved for further screening. Of these, 102 were excluded after being screened according to titles and abstracts. Of the 28 remaining articles, 16 studies were excluded due to inconsistency with the inclusion criteria set for the review. Finally, 12 studies that fulfilled the eligibility criteria were included for the systematic review and meta-analysis. General characteristics and descriptions of the studies selected for the meta-analysis were outlined in (Table 1)*.*

**Table 1:** Description of study participants and characteristics of Studies included in the systematic review and meta-analysis

S. No	Authors, Year	Study setting	Study design	Data collection methods	Sample size	Prevalence %	University/college	Out come	Specific factors
1	Marta T & Hinsermu B,2015 [19]	Institutional	cross-section	Structured questioners	624	11.4	Debere Markos university	Practice of emergency contraceptive	Age, Marital status, father’s educational status of the students and knowledgeable on EC
2	Wegene T & Fikre E, 2007 [17, 20]	Institutional	cross-section	Structured questioners	774	4.9	A.A& unity university	Practice of emergency contraceptive	Age, marital status and having child
3	Dejene T, TsionA et.al, 2010 [21]	Institutional	cross-section	Structured questioners	660	26.7	Adama university	Predictors of emergency contraceptive	Previous use of contraceptives, being married and age of 20 years and above, knowledge
4	Bahir K. A/Warri et.al, 2018 [22]	Institutional	Retrospective cross-section	Structured questioners	270	44.81	Jimma Teachers Training College	Practice of emergency contraceptive	Age and religion
5	Yohannes A, Hedija Y et.al, 2018 [18]	Institutional	cross-section	Structured questioners	515	78	Arbaminch university	Emergency contraceptive utilization	knowledge, good approach of EC service providers and positive attitude aboutECs
6	Kirubel M,Abebaw D et.al,2019 [23]	Institutional	cross-section	Structured questioners	241	33	Harar health science college	Practice of emergency contraceptive	knowledge
7	Bisrat Z, Bosena T et.al, 2015 [24]	Institutional	Cross-sectional	Structured questioners	489	46.3	Mizan-Tepi university	Emergency contraceptive utilization	Female students’ level of knowledge about EC, age at first sexual intercourse, previous use of regular contraceptives and history of pregnancy
8	Nigus C&Tilahun B,2010	Institutional	Cross-sectional	Structured questioners	508	30.9	Wollo university	Emergency contraceptive utilization	Currently, unmarried students and Those students who began sexual intercourse at age 13 years or less
9	Giziyenesh Kahsay, 2014 [25]	Institutional	Cross-sectional	Structured questioners	628	62.6	Aksum university	Emergency contraceptive utilization	Respondents who visited religious place at least once a week were single, respondents who have good knowledge on contraceptive and study year
10	Tewodros G, Tamene T et.al,2015 [26]	Institutional	Cross-sectional	Structured questioners	424	44.4	Wachamo university	Practice of emergency contraceptive	Ever married, good knowledge
11	Senait G/mariam,2012	Institutional	Cross-sectional	Structured questioners	331	13.1	Woliyta Sodo university	Practice of emergency contraceptive	Age, urban resident, ever had sex, favorable attitude
12	Habtamu A, Muleta M et.al,2014 [27]	Institutional	Cross-sectional	Structured questioners	549	18.4	Debere Markos university	Practice of emergency contraceptive	Age, ever married, favorable attitude

### Prevalence of emergency contraceptive practice

**Fig. 2:**
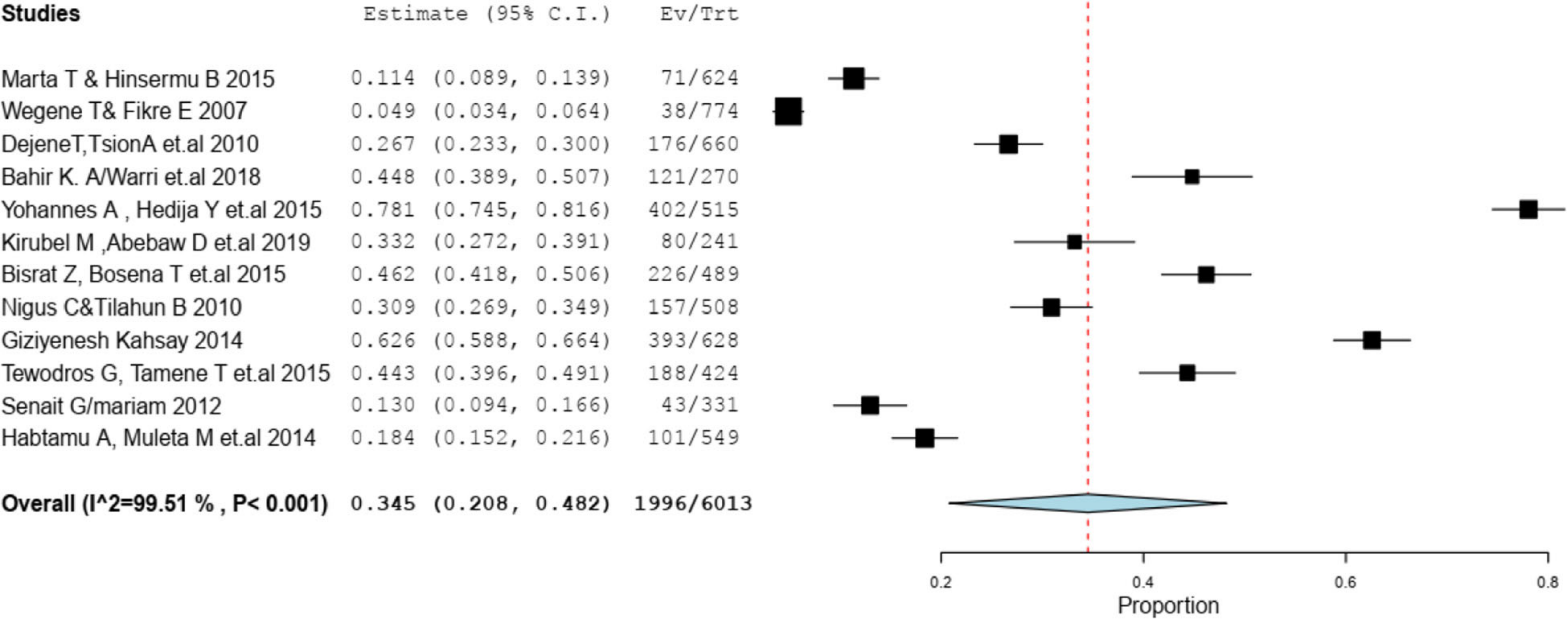
Pooled prevalence of Emergency contraceptive practice among female university students in Ethiopia, 2020

The pooled approximation of the magnitude of emergency contraceptive practice in Ethiopia was 34.5% [95% CI [20.8, 48.2%] (Fig. 2).

### Determinants of emergency contraceptive practice

The results of this review have shown determinants significantly associated with emergency contraceptive practice in Ethiopia were, Age [OR, 0.19; 95% CI:0.04, 0.98, *P* = 0.05] Previous contraceptive method use [OR, 0.22; 95% CI: 0.12, 0.40, *P* = 0.000001], Marital status [OR, 0.09; 95% CI: 0.02, 0.40, *P* < 0.002] and knowledge [OR, 0.12; 95% CI: 0.04, 0.37, *P* < 0.0003]. The review also verified that attitude was not a significant predictor of emergency contraceptive practice [OR, 0.61; 95% CI: 0.00, 136.12, *P* < 0.86].

### Age of the students

**Fig. 3:**
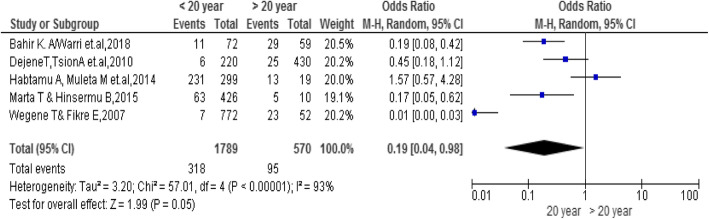
Association between the age of female university students with the emergency contraceptive practice among female students in Ethiopia 2020

The findings of the review indicated a significant association between age and the practice of emergency contraceptives. Female university students age less than 20 were 0.19 times less likely to practice emergency contraceptive compared to students who had age greater than 20 [OR = 0.19; 95% CI: 0.04, 0.98, *P* = 0.05]. Heterogeneity test indicated I2 = 93%, (Fig. 3).

### History of contraceptive method use

**Fig. 4:**
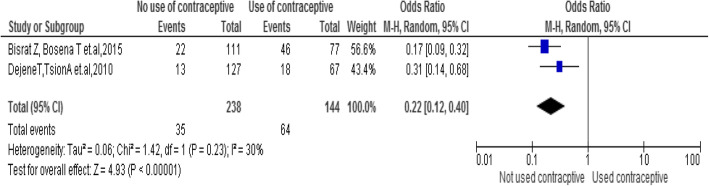
Association between a history of contraceptive methods use with the emergency contraceptive practice among female university students in Ethiopia, 2020

The findings of the review indicated a significant association between history of contraceptive method use and emergency contraceptive practice. Students who hadn’t have history of contraceptive methods use were 0.22 times less likely to emergency contraceptive practice compared to students who had a history of contraceptive methods use [OR = 0.22; 95% CI: 0.12, 0.40, *P* = 0.0001]. Heterogeneity test indicated I2 = 30%, (Fig. 4).

### Knowledge of the student

**Fig. 5:**
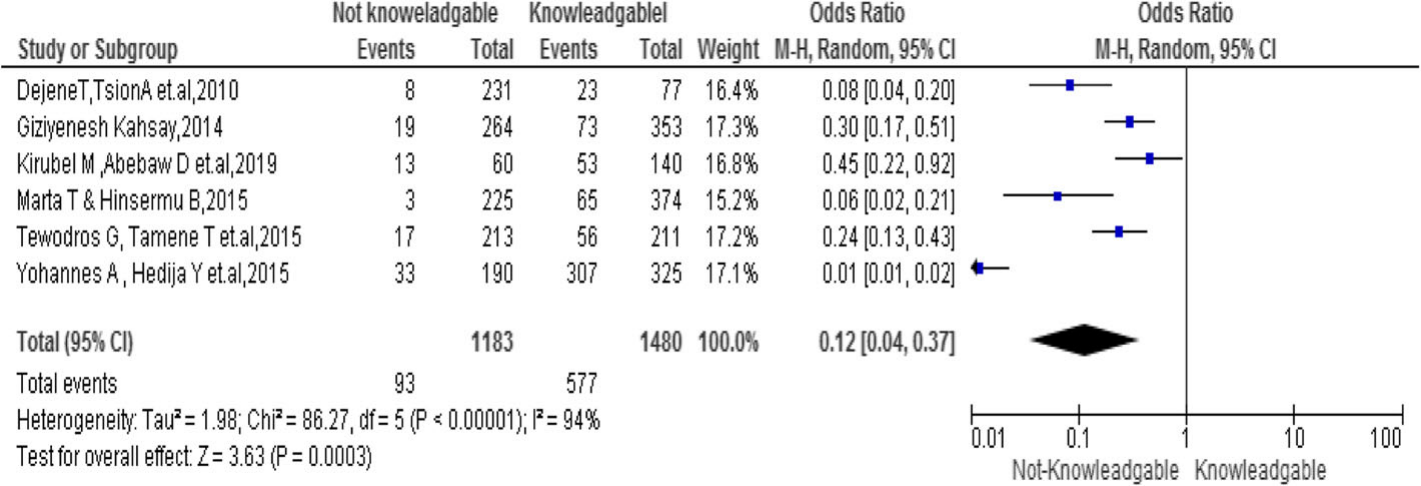
Association between Knowledge of students with the emergency contraceptive practice among female university students in Ethiopia,2020

This review demonstrated that there was significant association between students’ knowledge and emergency contraceptive practice in the random model [OR, 0.12; 95% CI: 0.04, 0.37; *P* = 0.0003]. Students who were not-knowledgeable were 0.12 times less likely to practice emergency contraceptives as compared to students who knew emergency contraceptives. But considerable heterogeneity was found too high (I^2^ = 94%), hence the random effect model was assumed in the analysis. Sensitivity analysis was done but did not bring significant change in the overall summary results of OR (Fig. 5).

### Marital status

**Fig. 6:**
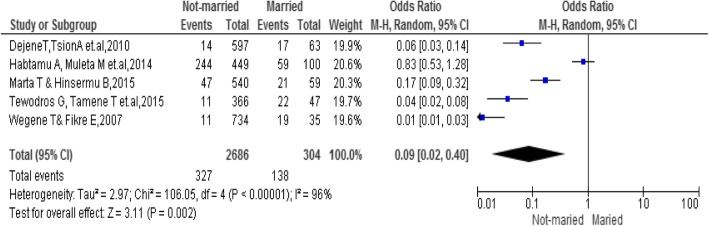
Association between marital status with the emergency contraceptive practice among female university students in Ethiopia,2020

Being married was significantly associated with the emergency contraceptive practice, the odds of emergency contraceptive practice were high among married as compared to others [OR, 0.09; 95% CI: 0.02, 0.40, *P* = 0.002]. Not-married students were 0.09 times less likely to practiced emergency contraceptives as compared to Married (Fig. 6).

### The attitude of the students

**Fig. 7:**
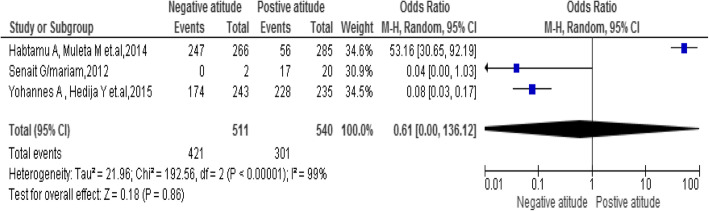
Association between attitudes of students with the emergency contraceptive practice among female university students in Ethiopia 2020

The results of the review presented there was no statistically significant association between attitudes of students and emergency contraceptive practice [OR, 0.61; 95% CI: 0.00, 136.12, *P* < 0.86]. The heterogeneity test was too high and the I^2^value was 93%. However, the investigators considered a random effect model for the analysis (Fig. 7).

### Publication bias

**Fig. 8:**
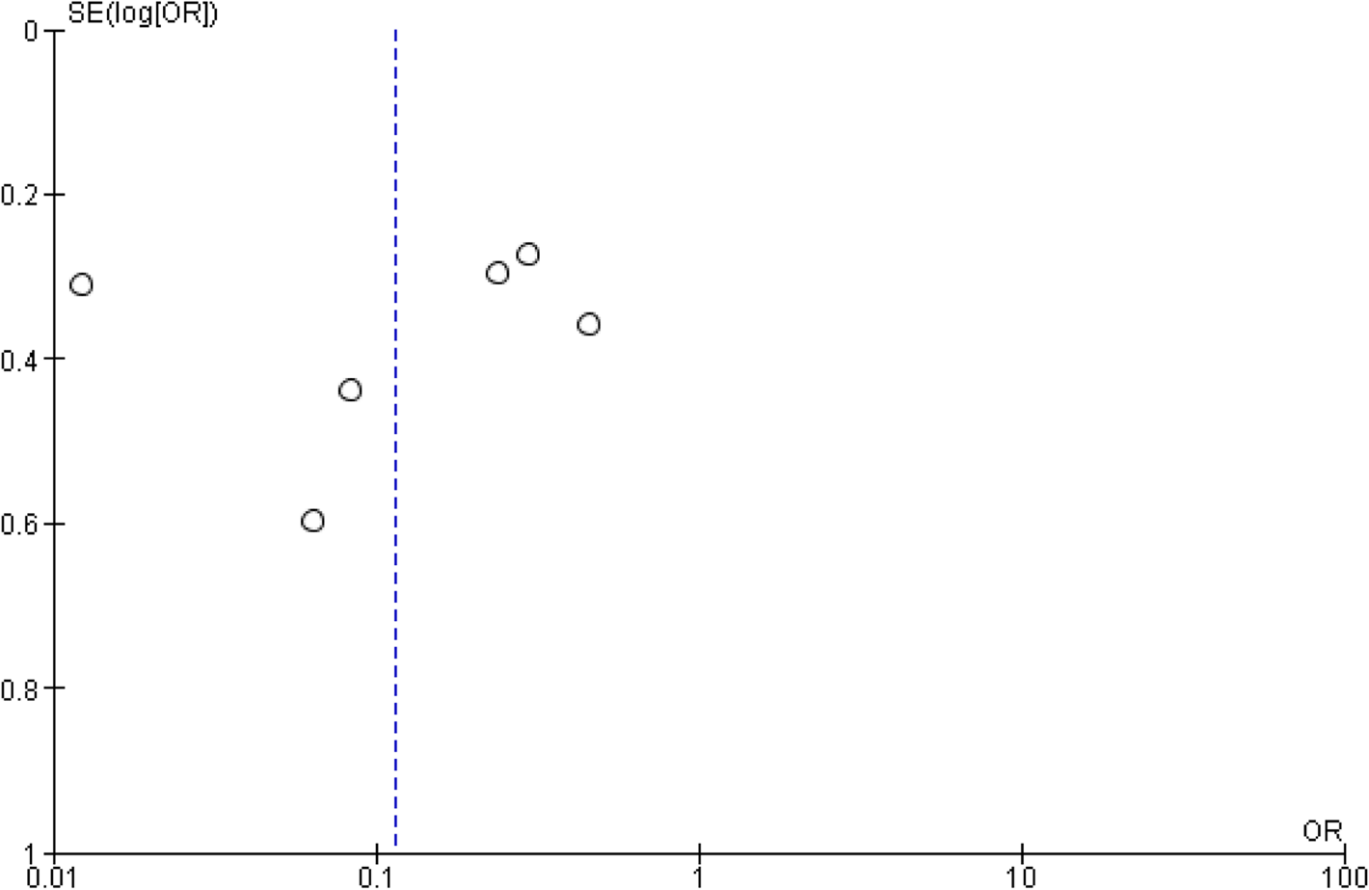
Publication bias on the knowledge of the students

**Fig. 9:**
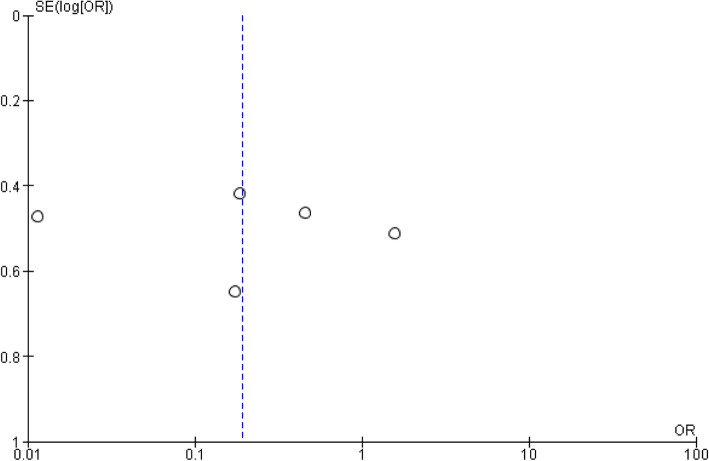
Publication bias on the age of the students

To check publication bias among the included studies for the meta-analysis, funnel plot and Egger’s test were carried out (Figs. 8 and 9).

## Discussion

Practicing emergency contraceptive to overcome unwanted pregnancy is a vital role, perhaps its practice should be under cautions. This comprehensive study provides potted information on inclusive determinants that limit the practice of emergency contraceptives in female university students in Ethiopia.

In this review, a total of 12 studies were included and all studies are collected primary data to assess the practice of emergency contraceptives. One of the breaches identified during this review was data were collected from students; which may be subject to recall bias. Our analysis revealed that the practice of students towards emergency contraceptives across the universities shows differences, in Arbaminch university 78% [18], Aksum university 62.8% [25] and Addis Ababa university 4.9% [17]. The possible reason for this discrepancy may be due to that both Arbaminch and Aksum university were tourist sites so that students might be exposed to unprotected sexual intercourse to overcome their soc-economic problem and in Addis-Ababa the awareness of the students was better due to the presence of many institutes in the compound over reproductive health.

This systematic and meta-analysis revealed that the overall prevalence of emergency contraceptive practice during the period studied in Ethiopia was 34.5% [95% CI [20.8, 48.2%]. This study is higher than findings from South African university students, 28% [10], Cameroon, 7.4% [11] Kenya, 20.2% [28], Kampala, 7.4% [29] Hong Kong, 12.9% [30]. The variation could be due to easy availability of the drug without prescriptions but lower than a study conducted in Federal Polytechnic Kaduna, Nigeria 38% [12], Ghana 41% [31]. The possible reason for this might be lack of awareness, lack of youth-friendly approach of the providers with in university clinics, less sexual experience and also poor knowledge of the students in all-rounded reproductive health issues.

In this review and meta-analysis, we found several determinants that have a significant association with emergency contraceptive practice in Ethiopia. In this review and meta-analysis, the emergency contraceptive practice was positively associated with work age, the previous history of contraceptive method uses, knowledge and marital status. The attitude was no association with emergency contraceptive practice.

The review revealed that students age less than 20 were less likely to practice emergency contraceptives [19–22, 27]. This finding was consistent with the finding of a study conducted in Kenya [28]. This might be related to, less exposure for sexual experience due to less adaptiveness for the environment and less sexual experience of their peer.

History of contraceptive method use is determinant that helps students to practice emergency contraceptives [21, 24]. This is because those students who have a history of contraceptive method use have better knowledge and awareness when compared with their counterparts.

Additionally, not-knowledgeable about emergency contraceptives was among the determinants contributing to the poor practice of emergency contraceptives in Ethiopia [19, 21, 23, 25, 26, 32]. This finding was similar to different studies [33, 34] This might be due to lack of awareness and lack of information.

The review revealed that students who hadn’t married were less likely to practice emergency contraceptives [19–21, 26, 27]. This finding was in line with Demographic and Health Survey reports of 21.7, 15, 11, and 10%, in Albania, Ukraine, Kenya, and Colombia, respectively [33, 34]. The possible reason for this might be less risk-taking behavior, lack of information and awareness.

## Conclusion

The emergency contraceptive practice among female university students in Ethiopia is 34.5%. This leads many students to discontinue their education with a lot of RH problems. So integrated effort is needed within Ethiopian ministry of education, minister of health and with respective higher institutions to avail family planning the course for every stream and need to strengthen reproductive health clinics and arranging service provision youth-friendly to extended the uptake to overcome the problem. Again, different mini-media clubs within institutions also incorporate the issue of RH as a major concern and take part in updating students. Age, marital status, knowledgeable and history of contraceptive use were among the determinants contributing under-utilization of emergency contraceptives.

### Limitation

Lack of study assessing the situation in all universities found in Ethiopia may have affected the generalizability.

## Supplementary information

**Additional file 1: Table S1.** Reporting Items for Systematic Reviews and Meta-Analyses: The PRISMA Statement Checklist.

**Additional file 2: Table S2.** Sample search string for CINHAL database, EBSCOhost Interface. **Table S3.** Sample search string for Medline database, EBSCO host Interface.

**Additional file 3: Table S5.** Quality assessment on included studies based on NOS checklist.

## Data Availability

If you request, we can avail all the data used.

## Abbreviations

CMA: Comprehensive meta-analysis
PRISMA: Preferred Reporting Items for Systematic Reviews and Meta-Analyses
JBI: Joanna Briggs Institute
