# Supplementary material for: Determinant of emergency contraceptive practice among female university students in Ethiopia: systematic review and meta-analysis
Source: Contracept Reprod Med. 2020 Oct 5;5:18. doi: 10.1186/s40834-020-00123-8 (PMC7534172; doi:10.1186/s40834-020-00123-8)
Supplement: Supplementary file 2 — Additional file 2: Table S2. Sample search string for CINHAL database, EBSCOhost Interface. Table S3. Sample search string for Medline database, EBSCO host Interface. [file 40834_2020_123_MOESM2_ESM.docx]

**Table S2: Sample search string for CINHAL database, EBSCOhost Interface**

| **#** | **Query** | **Limiters/Expanders** | **Last Run Via** | **Results** |
| --- | --- | --- | --- | --- |
| S4 | (Ethiopia) AND (S1 AND S2 AND S3) | Search modes - Find all my search terms | Interface - EBSCOhost Research Databases  Search Screen - Advanced Search  Database - CINAHL Complete | 6 |
| S3 | Ethiopia | Search modes - Find all my search terms | Interface - EBSCOhost Research Databases  Search Screen - Advanced Search  Database - CINAHL Complete | 967 |
| S2 | Emergency contraceptive practice/utilization | Search modes - Find all my search terms | Interface - EBSCOhost Research Databases  Search Screen - Advanced Search  Database - CINAHL Complete | 1432 |
| S1 | Practice OR Determinants OR Predictors | Search modes - Find all my search terms | Interface - EBSCOhost Research Databases  Search Screen - Advanced Search  Database - CINAHL Complete | 8796 |

**Table S3: Sample search string for Medline database, EBSCO host Interface**

| **#** | **Query** | **Limiters/Expanders** | **Last Run Via** | **Results** |
| --- | --- | --- | --- | --- |
| S4 | (Ethiopia) AND (S1 AND S2 AND S3) | Search modes - Find all my search terms | Interface - EBSCOhost Research Databases  Search Screen - Advanced Search  Database - MEDLINE | 2 |
| S3 | Ethiopia | Search modes - Find all my search terms | Interface - EBSCOhost Research Databases  Search Screen - Advanced Search  Database - MEDLINE | 3761 |
| S2 | Emergency contraceptive practice/utilization | Search modes - Find all my search terms | Interface - EBSCOhost Research Databases  Search Screen - Advanced Search  Database - MEDLINE | 3 |
| S1 | Practice OR Determinants OR Predictors | Search modes - Find all my search terms | Interface - EBSCOhost Research Databases  Search Screen - Advanced Search  Database - MEDLINE | 6093 |
