# Supplementary material for: Determinant of emergency contraceptive practice among female university students in Ethiopia: systematic review and meta-analysis
Source: Contracept Reprod Med. 2020 Oct 5;5:18. doi: 10.1186/s40834-020-00123-8 (PMC7534172; doi:10.1186/s40834-020-00123-8)
Supplement: Supplementary file 3 — Additional file 3: Table S5. Quality assessment on included studies based on NOS checklist. [file 40834_2020_123_MOESM3_ESM.docx]

**Table S5**: Quality assessment on included studies based on NOS checklist.

| **Study** | **1** | **2** | **3** | **4** | **5** | **6** | **7** | **8** | **9** | **Quality** |
| --- | --- | --- | --- | --- | --- | --- | --- | --- | --- | --- |
| Marta T & Hinsermu B,2015 |  |  |  |  |  |  |  |  |  | High |
| Wegene T& Fikre E,2007 |  |  |  |  |  |  |  |  |  | High |
| DejeneT,TsionA et.al,2010 |  |  |  |  |  |  |  |  |  | High |
| Bahir K. A/Warri et.al,2018 |  | × |  |  | × |  |  |  |  | Moderate |
| Yohannes A, Hedija Y et.al,2015 |  |  |  |  |  |  |  |  |  | High |
| Nigus C&Tilahun B,2010 |  |  |  | × |  |  | × |  |  | Moderate |
| Bisrat Z, Bosena T et.al,2015 |  |  |  | × | × |  |  |  |  | Moderate |
| Kirubel M ,Abebaw D et.al,2019 | × | × |  |  |  |  | × | × | × | Low |
| Senait G/mariam,2012 |  | × |  |  |  |  | × |  |  | Moderate |
| Tewodros G, Tamene T et.al,2015 |  |  |  | × |  | × |  |  |  | Moderate |
| Giziyenesh Kahsay,2014 | × | × | × |  |  |  |  | × | × | Low |
| Giziyenesh Kahsay,2014 |  |  |  |  |  |  |  |  |  | High |

N.B:

- **Yes = No=×**

1. Sampling frame appropriate to address the target population
2. Study population sampled appropriately
3. Sampling size adequate
4. Study subject and setting described well
5. Data analysis conducted sufficiently
6. Was valid methods used for identification
7. Condition measured in standard way
8. statically appropriateness
9. Was a response rate adequate? If not, low response rate managed appropriately
